# CART-analysis embedded in social theory: A case study comparing quantitative data analysis strategies for intersectionality-based public health monitoring within and beyond the binaries

**DOI:** 10.1016/j.ssmph.2020.100722

**Published:** 2020-12-17

**Authors:** Emily Mena, Gabriele Bolte, AdvanceGender Study Group

**Affiliations:** a University of Bremen, Institute of Public Health and Nursing Research, Department of Social Epidemiology, Bremen, Germany; b Health Sciences Bremen, University of Bremen, Bremen, Germany

**Keywords:** Germany, Health monitoring, Health reporting, CART-Analysis, Intersectionality, Sex/gender, Frequent mental distress, Mental health

## Abstract

Implementation of an intersectionality perspective into quantitative health research might support the process of unravelling complex socio-cultural and economic power relations which underly and shape patterns of health and disease within populations. Intersectionality-informed health monitoring and health reporting integrating a sex/gender-sensitive perspective could serve as a fertile ground to strengthen the essential function of health reporting to support political decision-making. We aimed at the integration of social theory into quantitative data analysis by taking into consideration 4 distinct central sex/gender theoretical concepts in health research. We developed and tested an intersectionality-based, sex/gender-sensitive strategy comparing 5 distinct models based on different combinations of the binary sex/gender variable, socio-cultural and economic variables (defined from an intersectionality perspective) as well as solution-linked sex/gender variables. We used CART-analysis as a quantitative, non-parametric, exploratory method to detect subgroups with high prevalence of frequent mental distress (FMD). Analyses were based on data from a National Health Telephone Interview Survey conducted in Germany. Depending on model and detected subgroup of our comparative approach, prevalence of FMD ranged between approximately 5 %–25%. Within the model including the binary sex/gender variable, socio-cultural and economic variables, sex/gender turned out to be the most important attribute. Comparing the models which included solution-linked sex/gender variables to the model not including these variables illustrated that the CART-algorithm was able to detect subgroups with the same prevalence of FMD, but with approximately 14% as opposed to 4.5% of the study population being affected. For these models, social support served as the primary splitting variable and not the binary sex/gender variable. Including or not including the binary sex/gender variable in the models with the solution-linked variables did not make a substantial difference. Embedding CART-analysis in social theory might have the potential to further sex/gender sensitivity in health reporting and might support decision-making when considering the allocation of health-related interventions.

## Highlights

• Suitability of recursive partitioning methods for intersectionality-informed and sex/gender sensitive health monitoring.
• Enhanced gender sensitivity by inclusion of modifiable solution-linked sex/gender variables into quantitative analysis.
• Identification of different subgroups with high prevalence of mental distress depending on applied theoretical gender concept.
• Detection of relevant subgroups affected by mental distress through sex/gender binary and solution-linked variables.

## Introduction

Differences in paid and unpaid labour are widely acknowledged as substantial causes for gender inequalities (Connell, 1987; Guinea-Martin, 2018), with women allocating two to six times as much of their day for unpaid responsibilities such as caregiving and housework compared to men (UN Women, 2018). Restrictive gender norms are recognized to sustain hegemonic masculinity by mainly disadvantaging women over men (Gupta et al., 2019; Sen & Östlin, 2007), but can as well affect the health of all sexes e.g. men, gender minorities or gender invariant persons (Hay et al., 2019; Heymann et al., 2019). Intersections of sex/gender with other socio-cultural and economic factors as socially assigned markers of power, such as age, race/ethnicity, education and sexuality, can as well shape different population subgroups of privilege and disadvantage. In turn, these patterns induce power dynamics and hierarchies between and within the groups of males and females or other categories of gender identity (Hay et al., 2019; Sen & Östlin, 2007; Commission of Social Determinants of Health (CSDH), 2008). The rising interest for implementation of an intersectionality perspective into quantitative health research (Bauer, 2014; Dubrow, 2008; Evans & Erickson, 2019; Seng et al., 2012) might reflect the increasing need for disentanglement of complex socio-cultural and economic structures which underly and shape patterns of health and disease within populations, to be able to understand and suitably address health inequalities.

The concept of intersectionality originated from black feminist theory. Focusing on categories such as sex/gender and race/ethnicity as distinct social categories was criticised by feminist scholars to not capture the unique living situations that African-American women would experience when marginalised because of being a woman *and* being black (Crenshaw, 1989, pp. 139–167). For a long time within health research, the concept of intersectionality was mainly implemented from a qualitative perspective but is progressively discussed and further developed in order to be adequately operationalised in quantitative research as well (Bauer, 2014; McCall, 2005). Grasping the complexity of social dimensions such as sex/gender, race/ethnicity, age, sexuality and education within a quantitative perspective is mostly achieved by taking an *intercategorical* approach to intersectionality (McCall, 2005) and without preferring one social dimension over another a priori (Veenstra, 2011). The *intercategorical approach,* unlike the *anticategorical approach*, temporarily upholds prevailing analytical categories in order to unravel relationships of inequality between various social groups and the underlying social structures along multiple and conflicting scales simultaneously (McCall, 2005).

In order to achieve meaningful evidence, a better linkage between health and social sciences and between research and policymakers is called upon (Gupta et al., 2019; Heymann et al., 2019; Weber et al., 2019). Against this background the collaborative research network AdvanceGender was founded. AdvanceGender consists of three projects, which aim to promote sex/gender-sensitive and intersectional quantitative health research and health reporting (Pöge et al., 2019). Within the research project AdvanceDataAnalysis we identify and develop strategies for intersectionality-based and sex/gender-sensitive quantitative analyses, which may be applied in health monitoring and reporting to support decision-making. Starting point of a project was a literature review (Mena & Bolte, 2019), which investigated how quantitative inter-categorical and intersectionality-informed analyses in health research were conducted. One result of this review was that intersectionality-informed multivariable analyses were mainly conducted in the U.S. and focused on the intersection between sex/gender and race/ethnicity by using them jointly as subgrouping variables and as parts of interaction terms in regression analyses. Only very few studies operationalised intersectionality from a simultaneous perspective using a non-parametric procedure such as CART-analysis by considering multiple intersectional-variables, without highlighting a specific predefined intersection a priori. Sex/gender sensitivity in the included studies was assessed by focusing on operationalisation of sex/gender, how sex/gender theories were used, and which central theoretical sex/gender concepts were referred to when aiming at explanation of intersectional sex/gender differences. A second result of this interview was that even though sex/gender was the main category of all included intersectionality-informed studies, it was exclusively operationalised as a binary with Gender being the most frequent theoretical sex/gender concept referred to when theoretically explaining sex/gender differences. None of the included studies had modifiable sex/gender aspects as a focal point.

The aim of the present case study was (1) to integrate social theory into quantitative data analysis by taking into consideration 4 distinct central sex/gender theoretical concepts in health research (Hammarström et al., 2014), and (2) to compare quantitative data analysis strategies without presuppositions about relevant, most affected subgroups. We applied CART-analysis (Breiman et al., 1984) as a non-parametric, exploratory method for data analysis which makes no assumptions about error distribution or independence (Lemon et al., 2003).

Mental health as a central public health issue (The Lancet, 2016), including evidence about highly significant sex/gender differences with regard to depression and anxiety (MSD 2002), was chosen as an exemplary thematic field. Besides the relevance for health monitoring and reporting, mental health is likewise meaningful from an intersectionality perspective: Minority stress as well as fundamental cause theory are predominantly considered, when focusing on disparities in mental health (Khan et al., 2017). These are anticipated to be a result of disadvantaging members of stigmatized minority groups through stressful experiences of discrimination (Khan et al., 2017; Meyer, 2003). Consequently, inequalities in mental health are likely to persist over time and replicate, on account of impeded access to resources such as knowledge, finances, political impact and social support (Khan et al., 2017). Frequent mental distress as measured and validated by the U.S. Department of Health and Human Services' Centers for Disease Control and Prevention served as the outcome of our case study.

## Methods

### Study population

This case study is based on the National Health Telephone Interview Survey ‘GEDA - German Health Update’ (GEDA 2009), which is part of the nationwide health monitoring in Germany carried out by the Robert Koch Institute (RKI). The GEDA study is a representative survey using computer-assisted telephone interviews, which is regularly repeated in German health monitoring and aims at the continuous observation of developments in disease and health as well as risk behavior. The random sample of telephone numbers were drawn according to the Gabler-Häder method (RKI, 2011). GEDA 2009 comprises health data for randomly selected German speaking adults aged 18–100 years (n = 21262) registered in Germany. The cooperation rate for participants, measured as the proportion of realised interviews with targeted persons that have been contacted, was 51.2% (RKI, 2011). Further details about design, methods and nationwide representiveness of the GEDA 2009 study population have been described elsewhere (RKI, 2012). The study population of the present analysis was defined as participants with available information about mental distress (n = 20760).

### Ethics and data protection

The data from the GEDA surveys are provided for public use and epidemiological research. In terms of data protection and informed consent GEDA 2009 was approved by The Federal Commissioner for Data Protection and Freedom of Information. Verbal informed consent was provided by all participants prior to the interview in GEDA 2009 (Lange et al., 2015).

### Health outcome for case study

Frequent mental distress (FMD) was assessed by one measure of the HRQOL-4, which has been developed and validated by the U.S. Department of Health and Human Services' Centers for Disease Control and Prevention. Study participants were asked the following question: “Now thinking about your mental health, which includes stress, depression and problems with emotions, for how many days during the past 30 days was your mental health not good?” (Moriarty et al., 2003). FMD in the present analysis is defined as 14 or more mentally unhealthy days in the past month reported by a respondent [versus 0–13 days]. The cut-off point of 14 days is based on its frequent use by clinicians and clinical researchers as a marker for clinical depression and anxiety disorders (CDC 1998).

### Binary sex/gender variable

Sex/gender was determined in GEDA 2009 by forced choice asking participants the question: “Are you male or female?” Information was given to interviewers about rationale for asking this question: “The question about gender is important because this determines which questions do not have to/should not be asked” (RKI, 2009).

### Socio-cultural and economic variables [intersectional variables]

From an intersectionality perspective we selected eight variables of GEDA 2009 capturing different socio-cultural and economic dimensions: Age [in years: 18–29, 30–39, 40–49, 50–59, 60–69, 70–79, 89+]; Education [categorized according to ISCED 1997 EU-classification: high/medium/low]; Occupational status [full-time, part-time, occasionally, not working], Professional status [blue-collar worker, white-collar worker, civil servant, freelancer, helping family, no profession, else]; Marital status [married, married - living separately, unmarried, divorced, widowed]; Disability status [yes, no]; Migration background (Schenk et al., 2007) [two-sided (non-German citizenship, respondent immigrated to Germany after birth or both parents not born in Germany), one-sided (one parent not born in Germany), no (without migration background)]; Urbanity/rurality [big city, city, rural, very rural].

### Sex/gender mechanisms variables [solution-linked sex/gender variables]

According to O'Campo and Dunn (2012) ‘solution-linked variables’ are variables that describe societal and contextual factors which underlie marginalization processes of socially defined groups based on unequal power relations but are modifiable factors and therefore public health relevant. We defined six variables of GEDA 2009 as solution-linked variables indicating possible mechanisms for development of sex/gender differences in health (Mena & Bolte, 2019; Pelletier et al., 2015): Family constellation [with partner and child(ren), with partner and no child(ren), no partner and with child(ren); no partner and no child(ren)]; Main earner status [main wage earner in the household: one person household, respondent herself/himself, partner, other, there is no main wage earner]; Perceived social support measured by the 3-item Oslo Scale (low, medium, high) (Dalgard, 1996; Meltzer, 2003); Burden due to household [5-point likert scale]; Burden due to childrearing [5-point likert scale]; Burden due to care [5-point likert scale].

### Statistical methods of an intersectionality-based, sex/gender-sensitive strategy

Analyses were based on data from all study participants of GEDA 2009 providing information about FMD (n = 20760), with a total sample of 9006 men and 11754 women. In order to detect subgroups with high prevalence of FMD classification task was performed with CART as decision tree building algorithm (Breiman et al., 1984) using the rpart package (Therneau and Atkinson, 2019) and R 3.6.1 (R Core Team 2013). CART-analysis has widely been used in health sciences and clinical research (Harper, 2005; Lemon et al., 2003). It is a binary recursive partitioning method for multivariable data, which can be used for non-parametric classification tasks, such as the detection of population subgroups that show higher prevalence with regard to a certain health outcome under study (Breiman et al., 1984). Compared to other parametric procedures, CART does not make distributional assumptions of any kind, either on the outcome or predictor variables and is not at all affected by outliers, collinearities, heteroscedasticity, or distributional error structures (Mubayi, 2017). Decision rules were selected by Gini impurity as a statistical measure of distribution, to compute the impurity of the data partitions (‘nodes’) with values ranging from zero to one. A value of zero indicates the lowest impurity and perfect classification (all participants within a node belong to the same class), a value of one indicates the highest impurity and even distribution (all participants within a node are randomly distributed). Cost weights were assigned to equally distribute sums of weights for cases and non-cases, thereby assigning equal importance to sensitivity and specificity (Cairney, 2013). Complexity parameter was set at 0.005 and minimum node size restricted to contain 1% of the respective analysis population. Fitted trees were ‘pruned’ according to the 1SE rule to develop a tree with the best size and lowest misclassification rate, by selecting the least complex tree whose error was one standard error above the tree with the smallest cross-validated error (Breiman et al., 1984). No other survey-specific weighing factors were applied. Unweighted percentage of population as well as prevalence of FMD were calculated for each node separately.

As intersectionality-based, sex/gender-sensitive strategy we considered different combinations of the binary sex/gender variable, socio-cultural and economic variables as well as solution-linked sex/gender variables in the CART analyses. Accordingly, we compared five different models in our case study:

- Model 1: binary sex/gender variable and intersectional variables;
- Model 2: binary sex/gender variable, intersectional and solution-linked variables;
- Model 3: intersectional and solution-linked variables, restricted to females only;
- Model 4: intersectional and solution-linked variables, restricted to males only;
- Model 5: intersectional and solution-linked variables.

With regard to the integration of social theory into analysis these 5 models may be linked to 4 central sex/gender theoretical concepts in health research focusing on sex, embodiment, gender equity, and gender equality. General definitions of the sex/gender theoretical concepts were mainly derived from Hammarström et al. (2014) and adapted for the present study, to further the translation of theory into statistical analysis as well as interpretation of results: The theoretical concept of ‘Gender’ is not restricted to a certain model, since it is considered in the models 2–5 by including solution-linked sex/gender variables. The binary sex/gender variable in model 1 refers to the theoretical concept of ‘Sex’ as an individual trait, classifying women and men based on their reproductive organs and functions (primarily sex as a binary classification on basis of socially rather than biologically defined cut-offs). The combination of the binary sex/gender variable with solution-linked variables refers to ‘Gender Equity’ as a needs-based approach for women and men (model 2). In this model the binary sex/gender variable is part of the analysis (in contrast to model 5) in order to allow for all other included variables to differ between the group of females and the group of males, according to the possibility of different needs of women and men. The approach of model 3 and model 4 might be interpreted as focusing on ‘Embodiment’ based on the health impact of internalised gender relations. In this context, the stratification by male/female might be understood as embodied gender. Distinguishing a group of females from a group of males could be understood as a result of bodies changing due to gendered environmental and behavioural factors. Model 5 is based on the theoretical concept ‘Gender Equality’ as ‘equal rights’ and thus absence of gendered discrimination. The underlying assumption is that if men and women are considered to be equal, the binary sex/gender variable as an attribute should not constitute a difference between individuals when e.g. developing a health promoting intervention and therefore should not be part of the multivariable analysis. Proportion of males and females within the identified subgroups of model 5 were calculated subsequent to the CART-analysis (shown in Fig. 3) to facilitate comparisons with other models including the binary sex/gender variable and in order to raise awareness about existing differences based on sex/gender inequalities.

## Results

The study population (n = 20760) comprised overall 10.6% persons with FMD. Prevalence in the male population (n = 9006) was 7.7%, prevalence in the female population (n = 11754) 12.8%, respectively. Table 1 shows the socio-cultural and economic (intersectional) variables of the study population and the prevalence of FMD by these socio-cultural and economic characteristics. Men in the study sample had more often a higher level of education, worked more often in full-time jobs, were more often blue-collar worker and less often white-collar worker in comparison to women. Across the categories of the intersectional variables, men had almost consistently lower prevalence of FMD than women in the study population. Highest prevalence for both groups can be found with respect to disability status, with 15.3% prevalence of FMD in men and 21.9% in women. Most pronounced absolute differences in prevalence of FMD between men and women occurred in the youngest age group and for participants having low education, a migration background or living in a very rural area. Very low absolute difference in FMD prevalence appeared for persons belonging to the highest age group, working only occasionally or who were widowed.

**Table 1:** Socio-cultural and economic characteristics of the study population and prevalence of FMD within categories of intersectional variables for the total study population and stratified by female/male.

	Total study population		Females		Males	
INTERSECTIONAL VARIABLES	Proportion of characteristic %(n)	Prevalence of FMD%	Proportion of characteristic %(n)	Prevalence of FMD%	Proportion of characteristic %(n)	Prevalence of FMD%
N	100 (20760)		56.62 (11754)	12.80	43.38 (9006)	7.71
AGE						
**18**–**29**	18.00 (3736)	10.65	16.39 (1926)	14.01	20.10 (1810)	6.76
**30**–**39**	16.13 (3349)	11.05	17.06 (2005)	13.01	14.92 (1344)	7.74
**40**–**49**	23.37 (4852)	10.59	23.61 (2775)	12.33	23.06 (2077)	7.88
**50**–**59**	17.40 (3613)	13.04	17.75 (2086)	14.43	16.96 (1527)	10.35
**60**–**69**	14.18 (2944)	8.53	14.06 (1653)	9.84	14.33 (1291)	6.25
**70**–**79**	8.34 (1732)	8.03	8.30 (976)	9.95	8.39 (756)	4.46
**80** +	2.57 (534)	10.30	2.83 (333)	8.68	2.23 (201)	10.38
EDUCATION						
**Low**	9.79 (2030)	14.24	11.40 (1338)	16.05	7.69 (692)	8.68
**Middle**	51.31 (10635)	11.73	55.24 (6481)	12.92	46.19 (4154)	9.13
**High**	38.89 (8060)	8.13	33.35 (3913)	10.21	46.11 (4147)	5.87
OCCUPATIONAL STATUS						
**Not working**	36.50 (7546)	12.76	40.99 (4796)	13.98	30.65 (2750)	9.20
**Full-time**	42.17 (8717)	8.91	27.96 (3271)	12.19	60.69 (5446)	6.76
**Part-time**	16.75 (3462)	10.46	25.88 (3028)	10.79	4.84 (434)	6.39
**Occasionally**	4.59 (948)	8.97	5.17 (605)	8.63	3.82 (343)	9.25
PROFESSIONAL STATUS						
**No profession**	8.03 (1658)	10.01	7.62 (890)	12.90	8.56 (768)	6.15
**Blue-collar**	15.90 (3282)	12.19	11.74 (1370)	15.74	21.32 (1912)	8.89
**White-collar**	55.51 (11456)	10.82	63.00 (7350)	12.29	45.78 (4106)	7.48
**Official**	7.44 (1536)	6.90	6.16 (719)	8.82	9.11 (817)	4.94
**Freelancer**	10.17 (2089)	8.82	7.83 (914)	9.62	13.20 (1184)	7.93
**Helping family**	1.01 (208)	8.17	1.53 (178)	7.07	0.33 (30)	13.33
**Else**	1.93 (398)	15.58	2.11 (246)	18.88	1.69 (152)	9.68
MARITAL STATUS						
**Married**	53.21 (11027)	8.82	52.34 (6142)	10.31	54.34 (4885)	6.52
**Married -living separately**	2.65 (550)	19.27	2.86 (336)	20.06	2.38 (214)	17.13
**Unmarried**	27.86 (5775)	10.42	23.97 (2813)	13.69	32.95 (2962)	6.99
**Divorced**	8.83 (1829)	15.86	10.46 (1227)	16.92	6.70 (602)	12.34
**Widowed**	7.45 (1544)	14.43	10.37 (1217)	13.41	3.64 (327)	13.51
DISABILITY STATUS						
**Yes**	8.09 (1677)	19.98	7.93 (931)	21.87	8.30 (746)	15.31
**No**	91.91 (19053)	9.76	92.07 (10809)	11.57	91.70 (8244)	6.87
MIGRATION BACKGROUND						
**No**	85.40 (17728)	10.05	85.30 (10025)	11.77	85.53 (7703)	7.25
**One-sided**	3.80 (789)	13.31	3.82 (449)	16.19	3.78 (340)	9.04
**Two-sided**	10.80 (2241)	13.92	10.87 (1278)	16.09	10.69 (963)	9.76
URBANITY/RURALITY						
**Big city**	31.39 (6457)	11.51	32.41 (3771)	13.22	30.06 (2686)	8.36
**City**	39.18 (8059)	10.22	38.73 (4507)	11.55	39.74 (3552)	8.02
**Rural**	15.40 (3168)	9.85	15.02 (1748)	11.82	15.89 (1420)	6.92
**Very rural**	14.03 (2887)	10.15	13.83 (1609)	13.26	14.30 (1278)	5.56

**Fig. 1:**
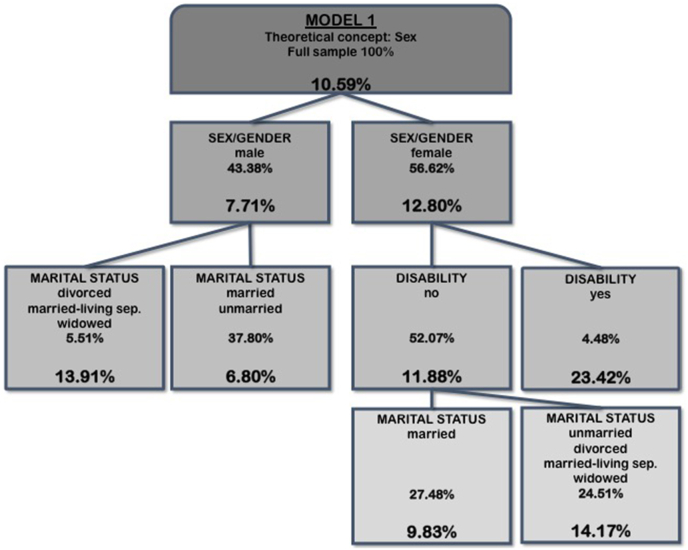
Splitting variables, proportion of study population and prevalence of FMD within subgroups (nodes) detected by CART-analysis based on binary sex/gender variable and intersectional variables of the full sample (Model1).

Model 1 (Fig. 1) included the binary sex/gender variable and intersectional variables. The first split was generated by sex/gender. In men, the highest FMD prevalence of 13.9% occurred for males who were married but living separately, were divorced or widowed. In women, the highest FMD prevalence of 23.4% was found for females with disability. For women without disability, the highest FMD prevalence of 14.2% occurred for the ones currently not being married. Lowest FMD prevalence of 6.80% was found for men who are married or still unmarried.

Table 2 shows prevalence of FMD within subgroups characterized by solution-linked variables, further stratified by females/males. Having a partner as main earner was the most common absolute difference between women and men, with 42.5% of the female study population versus 5.8% of the male study population. Women were more likely to be single-parent, to feel more burdened due to household, childrearing or care responsibilities or to have low social support compared to men in the study population. Women had consistently higher prevalence of FMD than men in all considered aspects. The female study population showed a prevalence around 20% or higher in those categories of the solution-linked variable, which characterized more disadvantaged living situations, except for main earner status: Being a single-parent, feeling strongly burdened by housework, childrearing or care activities or perceiving social support to be low. Men showed overall a similar pattern across these categories but had consistently lower prevalence of FMD compared to women. In men only one value of one solution-linked variable reached a prevalence of 20%, such as feeling strongly burdened by housework activities. Most pronounced absolute difference in prevalence of FMD when comparing women and men can be found for single-parenthood, being the main earner in the household or feeling strongly burdened by childrearing or care responsibilities, all aspects disfavouring women. Rather low absolute difference in prevalence between women and men was found with respect to having a partner as main earner or feeling more strongly burdened due to housework responsibilities. Relative difference in prevalence of FMD in women and in men are similar when comparing persons without children to persons feeling strongly burdened by childrearing responsibilities. Comparing persons without care tasks to persons feeling strongly burdened by care responsibilities, the relative difference in prevalence of FMD is higher in men.

**Table 2:** Solution-linked sex/gender characteristics and prevalence of FMD within categories of solution-linked variables for the total study population and stratified by female/male.

	Total study population		Females		Males	
SOLUTION-LINKED VARIABLES	Proportion of characteristic %(n)	Prevalence of FMD%	Proportion of characteristic %(n)	Prevalence of FMD%	Proportion of characteristic %(n)	Prevalence of FMD%
FAMILY CONSTELLATION						
**No partner, no child**	34.09 (6991)	12.34	32.89 (3824)	14.05	35.68 (3167)	9.39
**Partner, no child**	37.60 (7710)	8.86	35.26 (4100)	10.85	40.67 (3610)	6.14
**Partner, child**	24.49 (5021)	9.44	25.83 (3004)	10.70	22.72 (2017)	7.23
**No partner, child**	3.82 (783)	19.41	6.02 (700)	19.94	0.94 (83)	10.71
MAIN EARNER						
**1 Person household**	22.22 (4543)	13.74	22.83 (2643)	14.42	21.43 (1900)	11.43
**Respondent**	31.03 (6343)	9.32	16.13 (1868)	15.23	50.48 (4475)	6.59
**Partner**	26.56 (5430)	10.81	42.49 (4919)	10.66	5.76 (511)	9.92
**Another person**	8.85 (1809)	8.96	7.12 (824)	13.05	11.11 (985)	5.32
**None**	11.34 (2318)	9.19	11.44 (1324)	10.71	11.21 (994)	6.61
HOUSEHOLD BURDEN						
**Not applicable**	1.13 (235)	17.02	0.96 (112)	22.69	1.37 (123)	10.00
**No**	27.16 (5624)	8.09	23.00 (2696)	9.34	32.59 (2928)	6.51
**Rather no**	28.92 (5989)	7.91	25.18 (2952)	9.90	33.81 (3037)	5.74
**Moderate**	27.78 (5752)	11.25	30.99 (3633)	12.43	23.59 (2119)	8.42
**Rather yes**	8.42 (1743)	15.03	10.77 (1263)	15.28	5.34 (480)	13.04
**Yes**	6.59 (1364)	22.51	9.11 (1068)	22.33	3.30 (296)	20.00
CHILDREARING BURDEN						
**No children**	26.66 (5527)	11.16	25.53 (2997)	13.14	28.14 (2530)	8.12
**No**	39.05 (8096)	9.30	35.94 (4219)	10.83	43.12 (3877)	7.08
**Rather no**	13.44 (2786)	8.18	12.66 (1486)	9.85	14.46 (1300)	6.02
**Moderate**	11.38 (2360)	10.76	12.69 (1489)	11.33	9.69 (871)	9.23
**Rather yes**	5.06 (1049)	13.92	6.65 (781)	15.68	2.98 (268)	8.15
**Yes**	4.40 (912)	21.38	6.53 (766)	22.11	1.62 (146)	14.00
CARE BURDEN						
**No care tasks**	48.83 (10105)	10.30	49.66 (5818)	12.20	47.74 (4287)	7.12
**No**	35.52 (7352)	9.48	32.95 (3860)	11.18	38.89 (3492)	7.11
**Rather no**	5.86 (1213)	9.23	5.98 (701)	9.76	5.70 (512)	8.11
**Moderate**	4.53 (938)	13.22	5.03 (589)	14.78	3.89 (349)	9.52
**Rather yes**	2.54 (526)	14.83	2.95 (346)	15.49	2.00 (180)	12.57
**Yes**	2.72 (562)	24.20	3.43 (402)	25.48	1.78 (160)	18.18
SOCIAL SUPPORT						
**High**	34.51 (6894)	7.67	34.96 (3965)	9.46	33.91 (2929)	4.97
**Middle**	51.22 (10233)	9.38	50.56 (5733)	11.11	52.10 (4500)	6.70
**Low**	14.27 (2851)	21.64	14.48 (1642)	23.52	14.00 (1209)	16.91

**Fig. 2:**
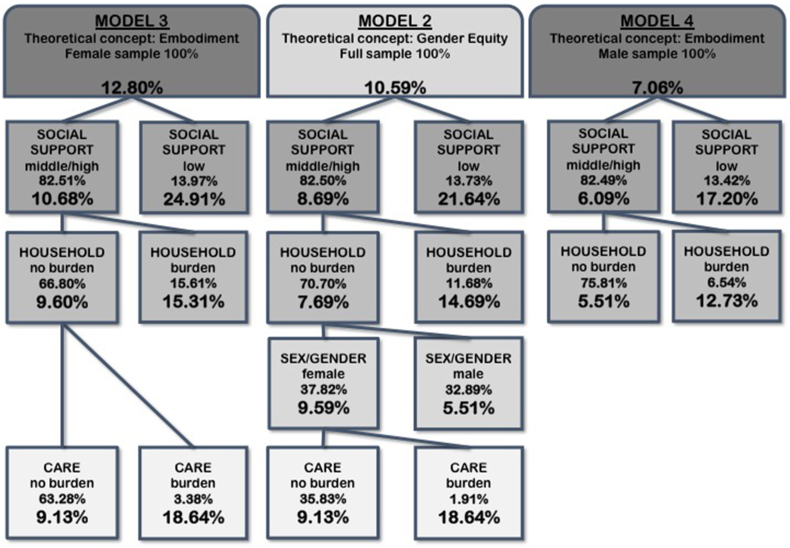
Splitting variables, proportion of study population and prevalence of FMD within subgroups (nodes) detected by CART-analysis based on binary sex/gender variable, solution-linked sex/gender variables and intersectional variables of the full sample (Model 2); based on solution-linked sex/gender variables and intersectional variables of the female sample (Model 3); based on solution-linked sex/gender variables and intersectional variables of the male sample (Model 4).

Model 2 (Fig. 2) included the binary sex/gender variable, intersectional and solution-linked variables. The first split was generated by social support. In persons with low social support, the highest prevalence of FMD was 21.6%. For study participants with middle to high social support the highest FMD prevalence of 14.7% can be found for persons who feel burdened by housework. The split for persons who do not feel burdened by household responsibilities is generated by sex/gender, with a FMD prevalence of 5.5% in men and 9.6% in women. Subsequently, FMD prevalence of 18.6% occurred in females who mainly feel burdened by care responsibilities. Lowest FMD prevalence was found for persons with middle to high social support, who do not feel burdened by household duties and are male 5.5%.

**Fig. 3:**
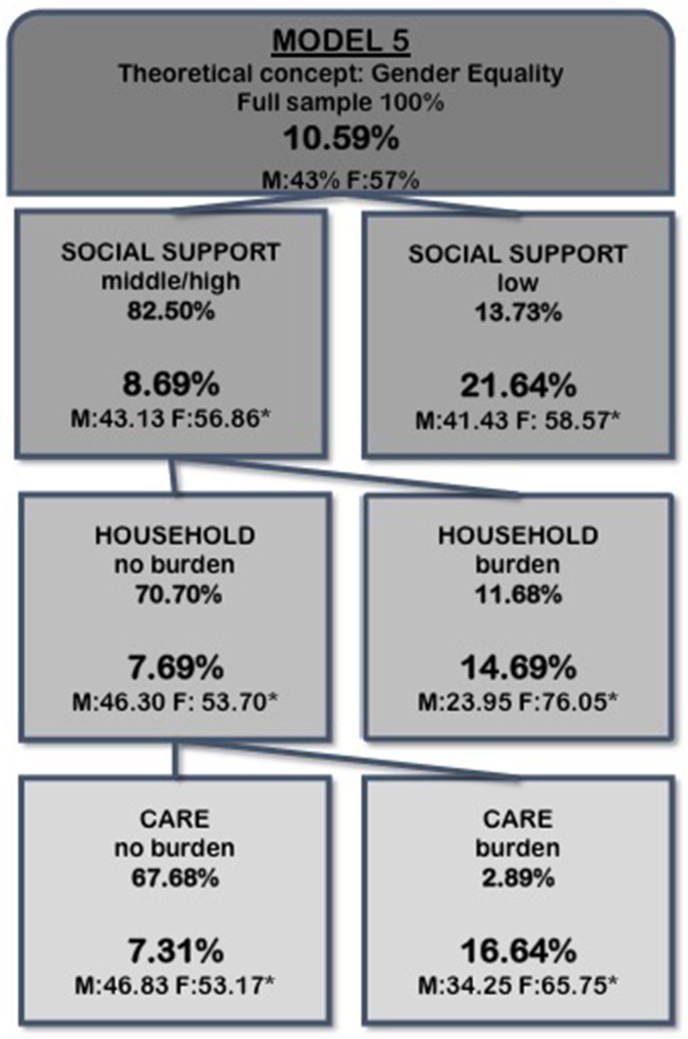
Splitting variables, proportion of study population and prevalence of FMD within subgroups (nodes) detected by CART-analysis based on solution-linked sex/gender variables and intersectional variables of the full sample (Model 5). *proportion of males and females within node.

Model 3 (Fig. 2) included intersectional and solution-linked variables of the female study population. The first split was generated by social support. In women with low social support, the highest prevalence of FMD was 24.9%. For women with middle to high social support the highest FMD prevalence of 15.3% can be found for females who feel burdened by housework. Women who perceive their social support to be middle or high, who do not feel burdened by housework, but feel burdened by care responsibilities had a FMD prevalence of 18.6%. Lowest FMD prevalence of 9.1% was found for females with middle to high social support, who do not feel burdened by household or care responsibilities.

Model 4 (Fig. 2) was restricted to males and included intersectional and solution-linked variables. The first split was generated by social support. In men with low social support, the highest prevalence of FMD was 17.2%. For men with middle to high social support highest FMD prevalence of 12.7% can be found for males who feel burdened by housework. Lowest FMD prevalence of 5.5% occurred for men with middle to high social support who do not feel burdened by household.

Model 5 (Fig. 3) was based on intersectional and solution-linked variables without the binary sex/gender variable. The first split was generated by social support. In persons with low social support, the highest prevalence of FMD was 21.6%. For persons with middle to high social support highest FMD prevalence of 14.7% can be found for those who feel burdened by housework. In persons who perceive their social support to be middle or high, who do not feel burdened by housework duties, but feel burdened by care responsibilities, prevalence of FMD was 16.6%. Lowest FMD prevalence of 7.3% was found for persons with middle to high social support, who are not burdened by household and care responsibilities.

## Discussion

In this case study we followed a comparative approach with 5 distinct models, each model including different combinations of the binary sex/gender variable, socio-cultural and economic variables as well as solution-linked sex/gender variables. We conducted CART-analysis with available data from a German health monitoring survey and included a wide variety of intersectional and solution-linked variables without a priori selection based on preconceptions. The 5 models were linked to 4 central sex/gender theoretical concepts in health research such as sex, gender equity, embodiment and gender equality. We considered the binary sex/gender variable in our analysis, though not always as starting point, and additionally integrated variables that may describe sex/gender relevant mechanisms that underlie societal power relations, by following a ‘solution-linked approach’ (O'Campo & Dunn, 2012). To allow for comparisons across the 5 models we focused our case study on mental health as a relevant health indicator for health reporting as well as intersectionality and sex/gender-based research. The models of our comparative approach allowed to detect different relevant subgroups contingent on the combinations of included variables. Moreover, the strategy enabled the identification of subgroups which would not have become visible by following a classical approach, e.g. only stratifying by one attribute such as female/male and not including solution-linked sex/gender variables. Depending on model and detected subgroup, prevalence of FMD ranged between approximately 5%–25%.

The main rationale of our case study was the development of strategies for intersectionality-based and sex/gender sensitive analysis within quantitative health research which could strengthen intersectionality-informed sex/gender sensitivity of health monitoring and reporting. By focusing on one specific intersectional variable such as sex/gender as the main axis of analysis, our strategy might be best classified as an intersectional perspective within an *unitary approach* as defined by Hancock (2007) or *intracategorical approach* as defined by McCall (2005). However, our explorative case study using CART-analysis for classification task can generally be described as intersectionality-informed since it mainly holds up with the 3 core tenets of intersectionality relevant to public health as proposed by Bowleg (2012). First, multiple social identities instead of a simple addition of social identities are considered when investigating privilege or disadvantage within different intersectional subgroups by using a non-parametric statistical procedure. Second, the detection of potentially marginalised intersecting groups is crucial for the analysis and in our case study furthered by the consideration of a large number of intersectional variables. In order to support the development of a sex/gender sensitive strategy for quantitative analysis, the binary sex/gender variable, distinct from other intersectional variables, was considered differently in sequential models. Together with specific solution-linked sex/gender variables, which were selected and conceptualised as possible mechanisms underlying sex/gender differences, all the sex/gender variables were set as the starting point of analysis. Third, interrelations of intersectional subgroups at the micro level with macrolevel structural factors are modelled by the integration of solution-linked sex/gender variables in order to illustrate disparate health outcomes in context of sex/gender discrimination.

To support decision making regarding allocation of health-related interventions, the explorative CART-models, based on different theoretical concepts of sex/gender and different combinations of intersectional and solution-linked variables, might be best compared and discussed with view on their ability to assist in the detection of meaningful subgroups. We considered FMD prevalence as well as proportion of the study population to characterize a “meaningful” subgroup for public health action. Comparing the subgroups across the models with the highest prevalence of FMD, models 2–5 including solution-linked sex/gender variables compared to model 1 not including these variables (theoretical concept: sex), differed substantially with regard to the proportion of the identified subgroups with 13.4 %–14.0% (participants with low social support) vs. 4.5% (women with disability status). Comparing results over all 5 models for men and women separately, most pronounced sex/gender differences in prevalence of FMD was found with regard to model 3 and model 4 (theoretical concept: embodiment). A direct comparison of model 3 and model 4 (theoretical concept: embodiment) most clearly illustrates how prevalence of FMD differs, in the group of men as opposed to the group of women, with higher prevalence of FMD for females regarding all values of the solution-linked sex/gender variables. In addition, burden due to care served as an important variable for the detection of a subgroup with higher prevalence of FMD only in model 3, which was based on the female sample, and not in model 4, which was restricted to the male sample. Finally, model 2 (theoretical concept: gender equity) shows how social support and burden due to household responsibilities are more relevant for the detection of subgroups with higher prevalence of FMD, than the binary sex/gender attribute. However, when comparing model 5 (theoretical concept: gender equality) with model 2 (theoretical concept: gender equity) the latter did not reveal a further 1% of males within the total study population, because the splitting generated by the sex/gender variable allowed only to detect higher prevalence of FMD in the female subsample with middle or high social support, who feel less burdened by housework duties, but feel strongly burdened by care responsibilities. Accordingly, model 5 (theoretical concept: gender equality) detected overall 2.9% of the study population with the same profile regarding intersectional and solution-linked variables as in model 2 (theoretical concept: gender equality), but beyond the sex/gender binary. Although we could only consider a binary sex/gender variable without further differentiation of other gender identities in our analyses, another possible advantage of model 5 (theoretical concept: gender equality) compared to models 1–4 might be, that it can be viewed as sex/gender sensitive for more than the binary differentiation, since sex/gender is an attribute that is only used for subsequent description and not part of the recursive partitioning process.

If our proposed analysis strategy is considered beneficial for health reporting, the explorative identification of subgroups with higher prevalence of FMD as well as larger proportion of the study population requires a thorough discussion about the interrelation of the outcome with the considered intersectional and solution-linked variables, the availability of theme-specific health monitoring data and the validity of the variables integrated into analyses. In order to not go beyond the scope of our case study, we did not explicate the source of power for each of the intersectional variables. A deeper understanding of the sources of power with regard to different socio-cultural and economic factors and their interrelatedness could further a more in-depth analysis and therefore appears appropriate for future studies. Nevertheless, putting results of our study into context would mean amongst other things to examine the direction of the causal relationship between the outcome and the exposure variables: Presumed that low social support was a result of but not a cause for higher prevalence of FMD, then defining social support as a solution-linked variable might be seen as critical, if decision-making regarding allocation of health-related interventions is based on information accessed through health monitoring and reporting. Eventually, our CART-analyses do not allow for straightforward assumptions about the direction of causation between mental health problems and perceived social support since they are based on exploratory analysis with prevalence data retrieved from a cross-sectional survey. Nevertheless, social support is generally acknowledged as a factor to reduce depressive symptoms (Meltzer, 2003; Piccinelli & Wilkinson, 2000; Turner, 1994), including substantial evidence based on prospective studies (Wang et al., 2018). Therefore, it appears plausible that social support served as the most important variable to detect meaningful subgroups in those CART-models which included solution-linked sex/gender variables.

In terms of research focused on the interrelation between social support and depression and the role of sex/gender, the higher rate of depression in women seems not to be explained by levels of social support (Piccinelli & Wilkinson, 2000). The more frequent observation of higher depression and analogously more social support in women when compared to men has actually been discussed as a ‘paradox’ (Turner, 1994). Perceived social support as measured by the 3-item Oslo Scale is meanwhile acknowledged as an important indicator related to positive mental health and therefore recommended for use in European health monitoring (Meltzer, 2003). The 3-item Oslo Scale was developed by Dalgard et al. (1995) and served as a measure of social support in the current analyses. Dalgard et al. (2006) aimed to address the described paradox with quantitative analyses based on reported data from several European countries, using the 3-item Oslo Scale. In reference to their results the authors concluded in line with Piccinelli and Wilkinson (2000), that the rate of depression was highest in women regardless of the amount of perceived social support.

The results of our analyses with social support as well as burden due to household and care representing the most important characteristics to identify meaningful subgroups with regard to FMD might serve as an impulse to further a discussion about the validity of the 3-item Oslo Scale from a sex/gender sensitive perspective. The 3-item Oslo Scale comprises items on primary support group, interest and concern shown by others, and ease of obtaining practical help (Meltzer, 2003). Social support in general is discussed as a construct of structural and functional constituents, with instrumental and emotional support as functional components relevant for mental health and depressive symptoms (Kocalevent et al., 2018). While the first 2 items of the 3-item Oslo Scale capture the structural dimension as well as emotional support, instrumental support is only reflected by the third item by asking how the participant perceives the availability of help from neighbours. Assuming that burden due to care and household responsibilities can be viewed as public health relevant indicators capturing a gendered lack of instrumental social support, the results of our analyses might be considered as a first indication to include these aspects into measures of social support recommended in health monitoring and reporting, or into epidemiological research focused on the interrelatedness of mental health, social support and sex/gender. The observed ‘paradox’ of higher depression and analogously more social support in women when compared to men (Turner, 1994) might be a result of not sufficiently considering and measuring gendered dimensions of instrumental social support when investigating the burden of mental illness in a population.

### Strengths and limitations

A limitation of our case study is the fact, that analyses were restricted to available data, that did not comprise information on gender identity or other possible differentiations apart from the sex/gender binary, which could capture the complexity of sex/gender more in detail. Respective information about study participants might be in part integrated in forthcoming surveys in context of health monitoring (Pöge et al., 2020). Limited data availability also concerns the included variety of intersectional and solution-linked variables. We checked all public use files with data of the adult German population, which are made available by the nationwide health monitoring carried out by the RKI. GEDA 2009, which can be viewed as a relatively old survey, provided the highest number of variables, that describe sex/gender relevant mechanisms. Future surveys could benefit from integrating more variables that may explain potential mechanisms that underlie societal power relations and discrimination, e.g. with regard to different socio-cultural and economic dimensions such as age, migration background and sex/gender. Another limitation concerns the possibility of same-source bias, which cannot be ruled out completely, especially when the interrelatedness of FMD with other solution-linked variables such as perceived social support and perceived burden due to household, childrearing and care responsibilities are of concern. According to Podsakov and Organ (1986) same-source bias is likely to be apparent, when overlap in variance of the measures themselves do not include the overlap in variance that is shared with the measurements’ referent domains. The overlap in variance of the measures might be a result of study participants adopting a coherent position when being asked to answer to a series of questions by giving each a summary judgement about their attitude. As stated in Podsakov and Organ (1986) this could encourage respondents to line up their judgments to a sequence of questions in accordance with prevailing lay theories on e.g. personality, behavior and psychological states (*consistency motif*). As a result, a substantive relationship between the predictor variables and the outcome might mistakenly be interfered based on artifactual covariance of the measures (Podsakov and Organ, 1986). However, Podsakoff et al. (2003)propose techniques for controlling same-source bias including cases where it is not possible to obtain alternative sources for the measured construct, which is principally the case with measures of self-perception. They advise to use different response formats for the measurement of the predictors as well as the outcome variable. Comparing the response formats of the measures used in the current study, it becomes apparent that the included measures do not contain similar items and that the response formats of the predictor variables are very distinct from the format of the outcome.

Intersectionality-informed quantitative analyses about study participation are scarce but an intersectional framework for the investigation of participation and representativeness of population-based studies has most recently been suggested in order to capture the multiplicity of systems of privilege and oppression in populations (Jaehn et al., 2020). Marginalised groups often choose not to participate in studies and furthermore interrelations of single aspects of social location with study participation are likely to differ when interrelated with other social dimensions (Larsson, 1994; Goldberg et al., 2001; Stang et al., 2005). We did not apply survey weights since they include information about weighting by the inverse probabilities of selection of only a few intersectional variables used in our case study such as age, binary sex/gender and education but not of all the others that we considered in our analyses simultaneously. Since the available survey weights do not capture the possibly complex interrelations between all the variables included from an intersectionality-informed perspective, which was one of the main objectives of our study, we restricted our analysis to unravel the complexity of FMD from an intersectional perspective only within the existing study population. Therefore, our results might only be generalizable to a certain extent when compared to the German population, since women as well as persons aged 18–24 years were overrepresented and individuals with migration background as well as persons aged 25–39 with low education were underrepresented in GEDA 2009 (RKI, 2011). However, we assume that higher participation of persons experiencing less privileged living situations might have put forth higher prevalence of FMD within subgroups detected by the CART-algorithm. Furthermore, overall prevalence of frequent mental distress might have been underestimated due to lower study participation rates of persons suffering from mental health problems (Francisco Perales & Bernard, 2018; Gayet-Ageron et al., 2011).

Strength of the present case study is our focus on a comparison of explorative models based on gender theory and intersectionality. One purpose of health monitoring and reporting is the identification of subgroups with higher burden of diseases. When considering the allocation of public health interventions, results from exploratory methods to identify subgroups differing substantially in prevalence of health outcomes, such as CART-analyses, may support policy makers decision-making (Lemon et al., 2003). In this respect, we tested CART-analysis (Breiman et al., 1984) as a method for our case study that might have the potential to build a bridge between research and policymakers, since its results can intuitively be interpreted (Morgan, 2014). Another strength of our study is that analyses are based on data from a national survey on health of adults in Germany and the inclusion of a fair amount of available intersectional and solution-linked sex/gender variables. Finally, the intersectionality-informed sex/gender-sensitive strategy might be adaptable to other socio-cultural and economic dimensions by setting another intersectional variable as the main axes of analysis.

## Conclusion

Even though the consideration of sex/gender at least as binary individual characteristic as standard approach in health reporting has been a substantial progress, a focus on stratification by the binary sex/gender variable might not sufficiently live up to the postulate of the concept of intersectionality. Health reporting might benefit from broadening the approach of data analysis from mainly stratification by sex/gender and age to a more explorative strategy as proposed in our case study. There is an increasing number of sex/gender specific as well as comparative reports which go beyond the scope of reporting statistics from a medical perspective and which integrate sex/gender related context and mechanisms into interpretation of the data. Therefore, it might be of particular importance to integrate sex/gender theory and intersectionality already in the underlying research process as put into practice in our case study, at least partly concerning the statistical analysis. Especially if decisions about allocation of public health interventions are central, our comparative approach might be a sex/gender sensitive way to detect larger population subgroups with high prevalence of health or disease within and beyond the sex/gender binary. Otherwise, when it comes to reporting results from intersectionality-based and sex/gender sensitive analyses with a narrower focus, then the gender equality perspective might be the most appealing, since it has a strong human-rights basis and is already considered to be relatively measurable and objective (Hammarström et al., 2014). In case the gender equality approach without the binary sex/gender variable but including solution-linked sex/gender variables is being followed, the subsequent characterization of the identified relevant subgroups by available data on sex/gender (binary variable or more comprehensive information on e.g. gender identity) is strongly recommended, in order to raise awareness about existing differences based on sex/gender inequalities. Since solution-linked variables such as social support, burden due to household, childrearing and care responsibilities can be assumed to represent modifiable aspects of societal power relations within and beyond the sex/gender binary, considering them in analyses with results aimed to support policy makers in decision-making might be a promising approach to reduce health inequalities.

## Data sharing statement

Datasets of the GEDA 2009 study are available for scientific use from fdz@rki.de.

## Ethics and data protection

The data from the GEDA surveys are provided for public use and epidemiological research. In terms of data protection and informed consent GEDA 2009was approved by The Federal Commissioner for Data Protection and Freedom of Information. Verbal informed consent was provided by all participants prior to the interview in GEDA 2009 (Lange et al., 2015).

## CRediT authorship contribution statement

**Emily Mena:** Conceptualization, Methodology, Formal analysis, Investigation, Writing - original draft, Writing - review & editing, Visualization, Project administration. **Gabriele Bolte:** Conceptualization, Writing - review & editing, Supervision, Funding acquisition.

## Declaration of competing interest

None declared.
